# What Do Effective Treatments for Multiple Sclerosis Tell Us about the Molecular Mechanisms Involved in Pathogenesis?

**DOI:** 10.3390/ijms131012665

**Published:** 2012-10-04

**Authors:** Katherine A. Buzzard, Simon A. Broadley, Helmut Butzkueven

**Affiliations:** 1 Department of Neurology, Royal Melbourne Hospital, Royal Parade, Parkville VIC 3050, Australia; 2 School of Medicine, Griffith University, Gold Coast Campus, QLD 4222, Australia; E-Mail: simon.broadley@griffith.edu.au; 3 Department of Neurology, Gold Coast Hospital, 108 Nerang Street, Southport QLD 4215, Australia; 4 Melbourne Brain Centre at the Royal Melbourne Hospital, Department of Medicine, University of Melbourne, Royal Parade, Parkville VIC 3010, Australia; E-Mail: butz@unimelb.edu.au

**Keywords:** multiple sclerosis, treatment, mechanism of action, immunomodulation

## Abstract

Multiple sclerosis is a potentially debilitating disease of the central nervous system. A concerted program of research by many centers around the world has consistently demonstrated the importance of the immune system in its pathogenesis. This knowledge has led to the formal testing of a number of therapeutic agents in both animal models and humans. These clinical trials have shed yet further light on the pathogenesis of MS through their sometimes unexpected effects and by their differential effects in terms of impact on relapses, progression of the disease, paraclinical parameters (MRI) and the adverse events that are experienced. Here we review the currently approved medications for the commonest form of multiple sclerosis (relapsing-remitting) and the emerging therapies for which preliminary results from phase II/III clinical trials are available. A detailed analysis of the molecular mechanisms responsible for the efficacy of these medications in multiple sclerosis indicates that blockade or modulation of both T- and B-cell activation and migration pathways in the periphery or CNS can lead to amelioration of the disease. It is hoped that further therapeutic trials will better delineate the pathogenesis of MS, ultimately leading to even better treatments with fewer adverse effects.

## 1. Introduction

Multiple sclerosis (MS) is a chronic, progressive, inflammatory disease affecting the central nervous system (CNS). Jean-Martin Charcot is generally credited with the first comprehensive clinical description of MS following his treatise “le sclerose en plaques” in 1868 [1]. Charcot identified patients who had discrete episodes of neurological dysfunction associated with inflammatory changes within the white matter of the brain and spinal cord [1]. MS is now recognized as a phenotypically heterogeneous disease affecting up to 1 in 1000 people in predominantly temperate areas, with a female to male predominance of 3:1 [2]. It is associated with significant morbidity and cost to the community due to the disease predominantly affecting young adults. MS has three subtypes; relapsing-remitting disease (RRMS) in which the disease exhibits a fluctuating course; primary progressive disease (PPMS) in which the course is inexorably progressive; and secondary progressive disease (SPMS) in which the course of the disease transforms from a relapsing-remitting to a progressive form.

Despite intensive investigation, the exact aetiology of MS remains elusive. It is well established that MS arises from an interaction between environmental and genetic factors. The human leukocyte antigen (HLA) complex has been estimated to contribute approximately 20% of the genetic risk to develop MS, with HLA-DRB*1501-DQB1*0602 (HLA-DR15) haplotype being the main allelic determinant [3]. Results from genome-wide association scans have identified further genetic loci associated with the development of MS. These include loci within the interleukin 2 receptor alpha (IL2RA), interleukin 7 receptor (IL7R) and cluster of differentiation 58 (CD58 or lymphocyte function associated antigen 3) [4,5]. It has been postulated that genetically susceptible individuals can develop MS following exposure to an as yet unidentified environmental trigger. The hypothesis is that, through molecular mimicry or epitope spreading, an abnormal immune response against self-antigens subsequently manifests in CNS inflammation, demyelination, axonal injury and progressive disability, features that comprise the pathological hallmark of MS.

Our understanding of the immunological processes potentially triggering MS has been greatly aided by the use of animal models such as experimental autoimmune encephalomyelitis (EAE). This model was derived from the immunization of mice with CNS homogenates. Nowadays, most laboratories use purified antigens to incite CNS inflammation, such as myelin basic protein (MBP), proteolipid protein, myelin oligodendrocyte protein (MOG) or synthetic peptides derived from these proteins [6]. However, the disease can also be induced by peptides derived from astrocytes or neuronal antigens, and some investigators have therefore suggested a closer resemblance to post-infectious or post-immunization acute disseminated encephalomyelitis [7]. Alternative methods for inducing EAE include the adoptive transfer of T cells isolated from mice primed with myelin antigens into naïve mice [8]. EAE models can display acute, relapsing and/or progressive neurological deficits associated with patchy CNS immune infiltration, sometimes associated with prominent demyelination, depending on the induction protocol [9]. EAE models have helped formulate the “T-cell hypothesis” of MS as an autoimmune disease caused by autoreactive T-cells targeted against CNS antigens.

**Figure 1:**
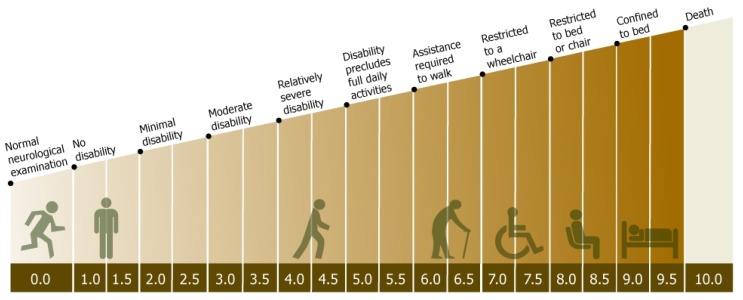
Schematic representation of the expanded disability status scale (EDSS).

**Figure 2:**
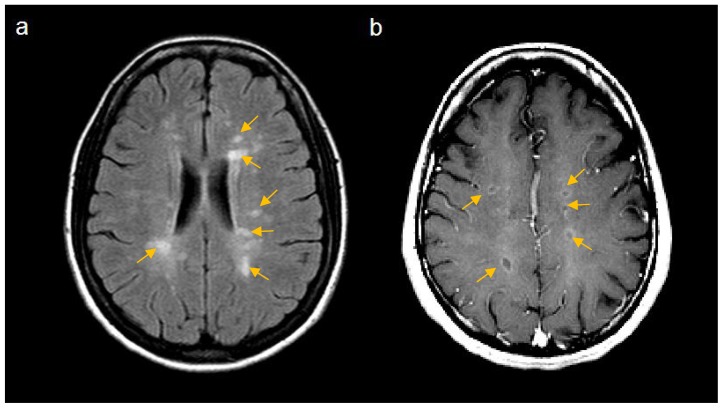
Axial magnetic resonance imaging showing (**a**) hyperintense lesions (arrows) on T2 weighted images and (**b**) ring, gadolinium (GAD)-enhancing lesions (arrows) on T1 weighted images.

In terms of assessing the efficacy of MS therapies, attention has primarily focused on three main measures of disease activity. For relapsing remitting disease, the frequency of attacks in the form of annualized relapse rate (ARR) has emerged as a useful concept. This involves calculating the mean relapse rate per year for the groups of patients being compared (typically active treatment against placebo over 1 or 2 years). Typical mean ARR for placebo treated groups have ranged from 0.33 to 1.28, with more recent trials generally enrolling patients with lower attack frequencies. Due to the random occurrence of relapses, measures of disability in the form of the Expanded Disability Status Scale (EDSS) can be difficult to assess, but another useful concept that has emerged is the notion of sustained disability progression over 3 or 6 months. In this concept transient changes in disability are ignored unless the deterioration is maintained for at least 3 or 6 months. Treatment arms are then compared by using the proportion free from progression after a period of time or in more sophisticated analyses using Kaplan-Meier curves and Cox’s proportional hazards ratios. The steps on the EDSS are indicated pictorially in Figure 1 but essentially this is a 10 point scale of disability in 0.5 point increments from normal (0.0) to death due to MS (10.0). The final paraclinical measure used relates to magnetic resonance imaging (MRI) activity either in the form of new T2 lesions (Figure 2a) [10] or new gadolinium (GAD) enhancing lesions on T1 weighted images (Figure 2b) [11]. The appearance of new GAD-enhancing lesions is thought to be consistent with breach of the blood-brain barrier in an acute lesion [12]. T2 lesions can be new (inflammation) or old (partial remyelination or gliosis).

MRI scans have become an essential component of clinical trials and are generally regarded as a good surrogate marker of disease activity [13]. The number of new lesions seen on MRI are significantly greater than the number of clinical relapses, indicating that most lesions (especially cerebral white matter lesions) are asymptomatic [14]. Thus, MRI is particularly useful in shorter early phase studies where there is often insufficient time to see significant differences in clinical measures of disease activity. Parameters used include total numbers of lesions (T2, T1 and GAD-enhancing), number of new lesions and volumes of lesions [15,16]. Latterly, measures of atrophy, including total brain volume have begun to be routinely included in analyses [17].

**Figure 3:**
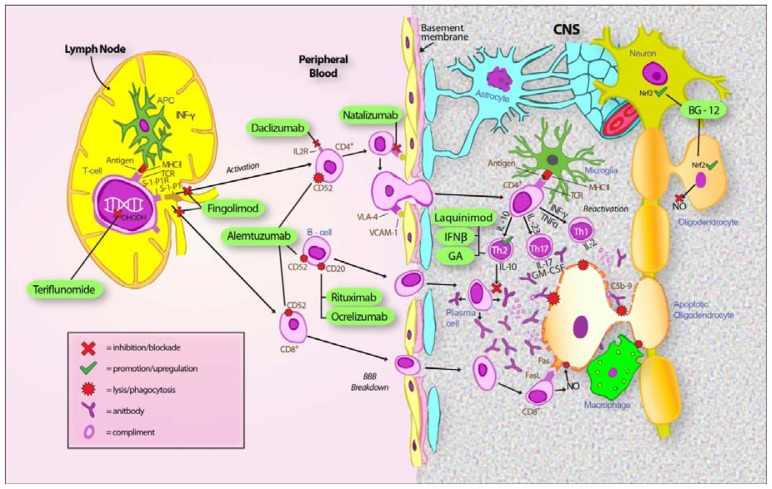
Schematic representation of multiple sclerosis (MS) pathophysiology indicating points of treatment intervention. APC antigen presenting cell; BBB blood brain barrier; C5b-9 complement complex 5b-9; CNS central nervous system; DHODH dihydroorotate dehydrogenase; FasL Fas ligand; GA glatiramer acetate; IFN interferon; IL2R interleukin 2 receptor; MHC I major histocompatibility complex I; NO nitrous oxide; Nrf2 nuclear factor (erythrocyte derived) related factor 2; S-1-P1 sphingosine-1-phosphate 1; TCR T cell receptor; VCAM-1 vascular cell adhesion molecule 1; VLA-4 very late antigen 4.

Extensive study of the pathology of MS and EAE have indicated a pattern of pathophysiology in which the activation of autoreactive T-cells and a breakdown of the blood brain barrier leads to infiltration of the CNS by inflammatory cells. Subsequent local reactivation leads to proliferation of various immune cell subtypes and initiation of the inflammatory cascade. This in turn leads to demyelination and axonal damage. This process is summarized in Figure 3.

### 1.1. T-Cells in MS

For several decades it has been recognized that CD4^+^ T-helper (Th) cells could be divided into functional subsets based on their ability to co-secrete specific groups of cytokines and to promote either cellular or humoral immunity [18]. Th1 cells produce pro-inflammatory cytokines such as interferon (IFN) γ and tumor necrosis factor (TNF) in response to stimulation by interleukin 12 (IL-12), thereby promoting cell-mediated immunity. In contrast, Th2 cells secrete IL-4, IL-5, IL-9 and IL-13, amongst others, which are involved in the development of humoral immunity. Each Th subset is capable of antagonizing the activities of the alternate subset [19]. Until relatively recently, the CNS inflammation seen in MS and EAE was thought to be mediated by autoreactive Th1 cells. This was despite conflicting evidence generated from some EAE models demonstrating that mice deficient in key Th1 cytokines had a worse disease phenotype [20,21]. Furthermore, anti-TNF treatment earned a warning from government agencies after studies showed these therapies could worsen or induce CNS demyelination [22,23]. These discordant findings began to challenge the hypothesis that abnormal Th1 activity was solely responsible for causing CNS demyelination in EAE and MS.

The discovery of an additional pathogenic Th subset, named Th17, led to a new hypothesis for the immune basis of MS. Th17 cells have unique effector functions, mediated via an array of proinflammatory mediators. IL-23 appears to be a key cytokine involved in the expansion of effector Th17 cells [24]. Recent studies in EAE have demonstrated that a key step in the proinflammatory actions of IL-23 is the stimulation of Th17-derived cytokine granulocyte-macrophage colony-stimulating factor (GM-CSF) [25]. Rather than acting directly on Th17 cells, GM-CSF is thought to promote the activation and recruitment of monocytes and antigen presenting cells to the CNS where they contribute to neuroinflammation [26]. Furthermore, GM-CSF participates in a positive feedback loop whereby it enhances the production of IL-23 from antigen presenting cells further sustaining the activation and maturation of Th17 cells [25]. Studies have previously demonstrated that GM-CSF is critical for the development of CNS inflammation in EAE [27]. This was again confirmed in a recent study demonstrating that autoreactive helper T cells deficient only in GM-CSF were unable to induce CNS inflammation in EAE [26]. Despite Th17 cells being named after their ability to produce IL-17, the role of IL-17 in CNS inflammation remains controversial. Some studies have demonstrated that removing IL-17, either by neutralizing antibodies or using knockout mice, reduces the severity of EAE and promotes recovery [24,28,29]. Others have shown that the absence of IL-17 signaling in IL-17 knockout mice does not completely prevent the induction of EAE in an adoptive transfer model [29]. A further study [30] has suggested that Th1 cells may still play a role in inciting CNS inflammation, given only Th1 cells can enter a non-inflamed CNS. Secretion of proinflammatory cytokines by activated Th1 cells may affect the expression of adhesion molecules such as vascular cellular adhesion molecule-1 (VCAM-1). Upregulation of VCAM-1 promotes vascular adherence of lymphoctyes via the binding of surface alpha-4 integrin. This would allow the secondary entry of pathogenic Th17 cells into the CNS where they could mediate inflammation and tissue damage [20]. Indeed, blocking the transmigration of lymphocytes into the CNS with monoclonal antibodies directed against alpha-4 integrin has proven to be an effective treatment in RRMS (see below).

The mechanism of Th17 cell mediated CNS inflammation has been the subject of intensive investigation. A model for illustrating the effector stage of Th17-induced EAE has recently been described [31]. In this model, it is hypothesized that myelin-specific Th17 cells in the periphery infiltrate the CNS, where they are reactivated by local antigen presenting cells. Reactivated Th17 cells undergo clonal expansion and secrete inflammatory cytokines such as IL-17, and this inflammatory milieu induces damage to myelin sheaths resulting in impaired nerve conduction. One potential mechanism by which this could occur is via astrocytes as intermediaries because IL-17 can stimulate astrocytes to upregulate inflammatory genes and thus proteins via actin 1 (Act-1) -mediated signaling cascades. Act-1 is a key adaptor protein in the IL-17 receptor signaling complex. The production of cytokines and chemokines from astrocytes secondarily promotes further recruitment of peripheral inflammatory cells leading to further tissue damage and lesion expansion [31,32].

Despite an emphasis on the role of CD4^+^ T-cells in the pathogenesis of MS, there is evidence that CD8^+^ T-cells may also play a role in the disease process. Data is emerging from GWAS studies identifying specific major histocompatibility complex (MHC) class I alleles that may predispose individuals to MS independently of the established susceptibility haplotype, HLA-DR15 [33]. Furthermore, support for a pathogenic role of CD8^+^ cells in MS has come from histological studies demonstrating that CD8^+^ cells outnumber CD4^+^ cells in acute and chronic MS plaques [34]. Indeed, the extent of axon damage in MS shows a significant correlation with the number of cytotoxic CD8^+^ cells in the same CNS tissue section [35]. These CD8^+^ cells are, potentially, clonally expanded T-cells rather than random CD8^+^ cells that have infiltrated into the CNS [34]. Like CD4^+^ cells, these CD8^+^ cells also reportedly express IL-17 [36]. It is interesting to note that several of the more effective treatments in RRMS, notably natalizumab, affect both CD4^+^ and CD8^+^ activity (see below). A potential synergistic role for both T-cell subsets was suggested by a recent study utilizing a MOG-reactive T-cell receptor (TCR) transgenic mouse that spontaneously expresses myelin-reactive CD4^+^ and CD8^+^ T-cells. The investigators demonstrated that the MOG-reactive CD8^+^ cells could independently induce a mild, delayed EAE, whereas their CD4^+^ counterparts incited severe EAE [37]. When both cell types were present, EAE was predominantly driven by myelin-reactive CD4^+^ cells [37]. It has been postulated that CD8^+^ cells are likely to require CD4^+^ cells for activation and memory formation in MS [34]. Following activation, it is hypothesized that effector memory CD8^+^ cells can subsequently infiltrate the CNS and stimulate macrophages and microglial cells, which in turn cause severe lesional tissue damage [34].

### 1.2. Regulatory T-Cells in MS

Whilst it is appealing to consider autoimmune disease as primarily a Th17-mediated process, this is likely to represent an oversimplification. Another subset of T-cells implicated in autoimmune disease are regulatory T-cells (T-regs). T-reg cells are a subset of CD4^+^ cells characterized by the immunophenotype CD25^+^Foxp3^+^. These cells have been implicated in suppressing autoreactive T-cells and promoting peripheral immune tolerance. Autoimmune disease may manifest following the failure of tolerance mechanisms thereby allowing the expansion of pathogenic T-cells. In a mouse EAE model, T-regs have been shown to suppress pro-inflammatory Th1 cytokine production and result in reduced disease severity [38]. The role of T-regs in MS is somewhat controversial with some studies showing the proportion of T-regs in MS patients is decreased compared to healthy control patients [39], whereas others report cell counts to be normal [40–42]. Others have proposed that T-reg effector functions are compromised in MS patients [43]. Some of the controversy over T-reg numbers in MS may be related to a degree of ambiguity over the markers used to identify this cell population or cell populations, as has been discussed previously [40].

### 1.3. NK Cells in MS

The latest immune cells implicated in autoimmunity are natural killer (NK) cells. NK cells are a heterogeneous group of T-cells characterized by the expression of the non-classical MHC class I molecule CD1d. NK cells play an important role in innate immunity including the targeting and removal of virally infected cells and tumor cells [44]. In humans, the majority of NK cells found in peripheral blood express low levels of the cell surface marker CD56. These NK CD56 dim cells mediate cytotoxicity of MHC class I-deficient cells via perforin and granzyme B. A much smaller percentage of peripheral blood NK cells are CD56^+^ bright cells. Following antigenic stimulation, NK cells with this immunophenotype have the capacity to produce vast quantities of cytokines, in turn influencing immune regulation [45]. They are also capable of localizing to secondary lymphoid tissue where there is the potential for interactions with autoreactive T-cells without prior specific sensitization.

Impaired NK cell function has been associated with a variety of autoimmune diseases, as well as potentially increasing an individual’s risk of developing some cancers and infections [46]. An early study suggested that impaired NK cell function may be involved in the pathogenesis of MS [47]. The potential importance of NK cells in both the induction- and effector-phases of MS was supported by the finding that antibody-mediated depletion of NK cells prior to immunization resulted in a more severe and relapsing form of EAE [48]. Over the last few decades, several groups have found NK cell defects in MS [46,49]. It remains to be determined whether these defects are responsible for the development of MS or rather, are a consequence of the disease process. Interest in the role of NK cells has been fuelled by recent studies implicating expanded NK cell populations in the mechanism of action of daclizumab and other MS treatments. These effects will be discussed in detail below.

### 1.4. B-Cells in MS

The dogma that MS is primarily a T-cell-mediated disease is being challenged by a growing body of evidence implicating B-cells as key effectors in the pathogenesis of MS. Histological studies of MS lesions frequently demonstrate a heterogeneous inflammatory infiltrate including T-cells, macrophages, B-cells and plasma cells. Whilst T-lymphoctyes and macrophages tend to dominate during active demyelination, it has been demonstrated that one of four pathological subtypes of active MS lesions is characterized by immunoglobulin and complement deposition [50]. These findings support a role for antibodies in mediating at least some of the CNS inflammation seen in MS [50]. Recent studies have identified ectopic lymphoid follicles resembling germinal centers in the meninges of patients with SPMS [51,52]. These structures contain proliferating B-cells as well as T-cells, plasma cells and dendritic cells. It has been postulated that clonally expanded B-cells originating in the meninges, may migrate to the parenchyma and participate in CNS damage [53].

An indicator that B-cells are involved in CNS inflammation in MS is the recognition that between 50–95% of MS patients have detectable oligoclonal bands restricted to the cerebrospinal fluid (CSF) early in the disease [54]. These oligoclonal bands represent antibodies, and intrathecal synthesis of clonal IgG is likely driven by the B-cell aggregates that populate the meninges and brain parenchyma in MS patients [53,55]. The exact antigenic target or targets of these antibodies remain unclear. Many studies have sought to identify specific antigens, either infectious agents or CNS antigens, with inconsistent results [56]. A recent study has disputed previous finding that antibodies derived from the CSF of MS patients could react against CNS white matter tissue from MS patients [57], concluding that the oligoclonal B-cell response in the CSF of MS patients is not directed against known myelin antigens [57]. There are similarly conflicting results regarding antibody-mediated demyelination in MS. An early study identified specific myelin protein autoantibodies bound to disintegrating myelin around axons in acute MS lesions [58]. In contrast, several studies have now demonstrated that some CNS autoantibodies can promote remyelination depending on the microenvironment [59].

MS is recognized as a disease that manifests as a result of genetic and environmental factors. Whilst the strongest genetic risk is associated with the HLA-DR15 haplotype, a large number of other loci carrying lower disease susceptibility have been identified. These include several loci within genes known to be involved in B-cell function such as CD40 and chemokine receptor 4/5 [60]. Over the years, considerable research effort has gone into identifying possible environmental triggers for MS. The association between geographical location and the risk of developing MS has focused attention on the possibility of an infectious agent as the inciting factor in MS. Epstein-Barr virus (EBV) shows the most promise in terms of an association with MS. EBV is a member of the human herpes virus family that exists as a latent infection in B-lymphoctyes [61]. The prevalence of EBV infection in the general population is approximately 90–95%. However, MS patients are almost universally (>99%) infected [62]. The relative risk of MS following infectious mononucleosis has been calculated at 2.3 (95% CI, 1.7–3.0) [63]. Indirect evidence supporting a role for EBV in the pathogenesis of MS comes from epidemiological studies showing an association between increasing levels of EBV nuclear antigen 1 antibodies (EBNA-1), a marker of the latent phase of the virus, and risk of developing MS, with the serum samples obtained five or more years prior to the development of MS [62]. EBNA-1 IgG levels also correlate with the development of GAD-enhancing lesions in MS, thereby strengthening the association between EBV or the immune response against it, and MS disease activity [64]. It has been proposed that, in genetically susceptible individuals, expanded EBV-infected autoreactive memory B-cells could migrate to the CNS and promote inflammation via the production of pathogenic autoantibodies and the stimulation of T-cells [65]. This hypothesis has also been linked to vitamin D deficiency, given vitamin D-deficiency can result in decreased numbers of cytotoxic CD8^+^ T-cells, potentially reducing the surveillance for virally infected cells and allowing the expansion of EBV-infected autoreactive B-cells [66]. Latent EBV infection may also participate in CNS inflammation in active MS lesions by stimulating IFN-α production from innate immune cells [67]. Attempts to identify EBV-infected cells in the brains of MS patients have been disappointingly inconsistent [68–70]. Rather than directly infecting CNS tissue, it has been hypothesized that EBV infection may lead to a cross-reaction to myelin antibodies via “molecular mimicry” [71]. It is known that infection of B-cells with EBV leads to immortalization [72] and it has been hypothesized that immortalization of autoreactive B-cells may lead to autoimmunity in MS and other autoimmune diseases [65].

A recently published review provides evidence that markers of B-cell presence and activation correlate with disease activity and progression in MS [60]. The effect of B-cell depletion on disease outcomes in murine models of EAE is highly variable and appears to depend on the B cell depletion strategy employed as well as the EAE induction protocol [73]. In contrast, clinical trials of anti-CD20 (rituximab) therapy in RRMS have consistently shown strong clinical benefit [74–76]. These positive results occurred without any significant effect on plasma cells or serum and CSF antibody titres, thereby supporting a role for B-cells in the pathogenesis of MS, independent of antibody production. The above raises the question of the other potentially pathogenic roles for B-cells. It has been proposed that activated B-cells may act as antigen presenting cells in MS [77]. In conjunction with co-stimulatory molecules, antigen presenting B-cells could therefore help polarize the T-cell response towards either Th1 or Th2 pathways. Alternatively, aberrant B-cell responses may directly stimulate T-cells through the production of pro-inflammatory cytokines; a process referred to as “bystander activation” [78]. In contrast, increased B-cell production of the anti-inflammatory cytokine, IL-10, can downregulate T-cell activity thus promoting disease resolution [73]. Clearly the role of B-cells in MS is complex with growing evidence that B-cells can manipulate T-cell responses to favor either a pro-inflammatory (pathogenic) or anti-inflammatory (regulatory) state. These divergent immunomodulatory roles for B-cells have implications for B-cell depleting therapies in MS, as will be discussed below.

### 1.5. Monocytes and Macrophages in MS

Macrophages and their native CNS counterparts, microglia constitute just 12% of the cells of the CNS [79]. Although their role in lesion pathogenesis is uncertain, macrophages derived from peripheral blood monocytes are important mediators of MS-related CNS injury. Specifically, macrophages and microglia are thought to be the main cell type responsible for lesional and perilesional axon killing [35,80,81]. As CNS axons do not regenerate in humans, this represents the most likely pathogenic mechanism for disability progression in progressive forms of MS. Macrophages constitutively express MHC class II and microglia can be induced to express MHC class II in response to IFN-γ, lipopolysaccharide, adhesion molecules and IL-12 [82]. Pathological and human in-vitro studies of microglia indicate an antigen presentation role in MS [83,84]. Activated macrophages also synthesize and release TNF-α and nitrous oxide thereby leading to oligodendrocyte and axon damage [85,86]. Finally, macrophages have been demonstrated to actively phagocytose myelin in MS lesions [87,88]. This final step in the process may occur via membrane Ig Fc receptors on macrophages interacting with opsonized C3 complement binding to 29,39-cyclic nucleotide 39-phosphodiesterase, a component of the oligodendrocyte membrane [89].

### 1.6. Through the Looking Glass

Current and emerging treatments for MS have been generated from our presumed understanding of the immune processes involved in MS. Much of this knowledge has come from animal models of EAE, which, whilst they share some clinical and pathological similarities with MS, by no means reflect the complexities that are likely to underlie the disease in humans. The early recognition that MS appeared to be driven by autoreactive Th1 cells directed treatment strategies towards those that could manipulate T-helper subsets away from pro-inflammatory Th1 and Th17 pathways towards the more anti-inflammatory Th2 subset. Limited success with initial MS treatments drove the search for novel immune targets. Preventing the entry of inflammatory cells into the CNS compartment proved to be an extremely valuable immunomodulatory strategy in MS, however increased efficacy appeared to come with significant risk of adverse effects in the form of progressive multifocal leukoencephalopathy [90]. Manipulating the immune system by targeting individual cytokines or cell surface markers continues to be an appealing strategy, although unpredictable and unexpected results remind us (e.g., TNF-α blockade [23]) that the immune system is complex and cannot easily be dissected into discrete and well-defined pathways. The purpose of this review is to explore the mechanisms of action of current and emerging MS treatments in an attempt to gain further insight into the pathogenesis of MS. We have not covered acute treatments for MS (e.g., corticosteroids, plasmapheresis) nor have we included non-specific immunosuppressive treatments such as azathioprine, mitoxantrone or bone marrow transplantation. The evidence for efficacy with these therapies is less secure (except for mitoxantrone in SPMS), they come with significant potential for adverse effects and their modes of action are sufficiently broad that they do not shed any significant light on the pathogenesis of MS.

Table 1 gives a list of the treatments considered in this review together with a summary of their efficacy and adverse effects. The presumed mechanisms of action for these treatments are summarized in a schematic of the pathophysiology of MS in Figure 3.

## 2. Approved Disease Modifying Treatment in RRMS

### 2.1. Interferons

Type 1 interferons (IFNs) have been the mainstay of MS treatment for around 20 years. Interferon-β (IFNβ) reduces the rate of MS relapses by a third when compared to placebo [14,91]. Despite this relatively modest effect on relapse rates, long term data and historical comparisons suggest that their effect on progression may be quite significant over decades [106]. However, a recent study has called into question the presumed long term efficacy of IFNβ [107]. The arrival of newer and more effective MS treatments has led to a plateauing of their use but the strength of IFNβ lies in the knowledge that it is a safe long-term treatment.

Endogenous type-1 IFNs have long been recognized as important players in the induction of cellular resistance to viral infections, acting primarily via IFN-αβ [108]. Over the years, a variety of other anti-inflammatory effects of type-1 IFNs have been recognized [109]. These properties have been exploited in the treatment of RRMS. One of the earliest findings was that treatment with type-1 IFNs could divert the cytokine milieu towards an anti-inflammatory phenotype. Elevated levels of anti-inflammatory mediators such as IL-10 and IL-4, were demonstrated in cell lines and immune cells derived from MS patients treated with type-1 IFNs [110–112]. These anti-inflammatory effects appeared to coincide with a reduction in pro-inflammatory mediators such as IL-17, IL-23 and osteopontin [113–117]. The effect of type-1 IFNs on cytokine production may be mediated by direct actions on T-cells or via effects on innate immune cells such as macrophages and dendritic cells [118]. Regardless of the exact mechanism, it is now generally accepted that type-1 IFNs can reduce CNS inflammation in MS by diverting the immune system away from a pro-inflammatory Th1 and Th17 pathway toward a more anti-inflammatory Th2 subset.

A further mechanism of action for type-1 IFNs in MS may involve the expansion of regulatory cells such as NK cells and T-regulatory cells (T-regs). Several human studies have now demonstrated that IFNβ-1a treatment results in the expansion of NK CD56^+^ bright cells [119–122]. In one study, this effect correlated with a positive clinical response [122]. Further studies are required to fully elucidate the role of NK cells in the treatment of MS, but their potential as a disease marker will be further discussed in relation to daclizumab therapy.

Dysfunctional T-reg activity has been implicated in the pathogenesis of MS [40]. Several studies have shown enhanced T-reg function in RRMS patients treated with IFNβ-1a [123,124]. It has also been demonstrated that IFNβ-1a increases the proliferation of T-reg cells through upregulation of the glucocorticoid-induced TNF receptor ligand found on dendritic cells [125]. IFNβ-1a treatment also reduced the expression of cytotoxic T-lymphocyte antigen 4 on T-reg cells which in turn increased the responsiveness of T-regs to stimulation [125]. Together, these mechanisms promote the proliferation of anergic T-reg cells and enhance the suppression of autoreactive T-cells.

In addition to the effects on immune cells, type-1 IFNs have also been shown to affect a variety of adhesion molecules and matrix metalloproteinases which in turn can impair the migration and trafficking of lymphocytes into the CNS [118]. Additional putative mechanisms of action for type-1 IFNs may include the induction of nerve growth factors (NGF) with several studies demonstrating significantly increased levels of NGF levels in brain endothelial cells, peripheral blood mononuclear cells and mouse astrocytes treated with IFNβ [126–128]. These latter findings would support a possible role for IFNβ in aiding CNS repair and recovery.

The understanding that B-cells may be important in the pathogenesis of MS is rapidly gaining momentum in light of the reported benefits of monoclonal B-cell antibodies in attenuating disease. A recent study has characterized the effect of type-1 IFNs on B-cells in RRMS patients [129]. This study demonstrated that IFNβ-1b inhibited B cell stimulatory capacity by suppressing CD40 and CD80 expression. This suppression in turn impaired antigen-specific T cell proliferative responses. IFN treatment also inhibited B-cell secretion of pro-inflammatory cytokines such as IL-1 beta and IL-23 whilst stimulating the secretion of the anti-inflammatory cytokines IL-12 and IL-27. Presumably via this modulation of cytokine secretion, supernatants from IFNβ-1a treated B-cells inhibited Th17 cell differentiation [129]. The effect on B-cell function may represent yet another mechanism for which type-1 IFNs exert their therapeutic effect in RRMS.

A somewhat intriguing finding is that type-1 IFNs are not universally beneficial in autoimmune diseases and IFNβ appears to precipitate antibody mediated autoimmunity (Graves’ disease and systemic lupus erythematosus) in some individuals [92,93]. Only 30–50% of MS patients appear to do well with type-1 IFN treatment [130] and the closely related antibody-mediated demyelinating disease, neuromyelitis optica (NMO), may be aggravated by type-1 IFN treatment [131]. Following work in animal models it has recently been postulated that type-1 IFNs may be immunomodulatory in Th1-mediated diseases but pro-inflammatory in diseases driven by Th17 pathways [132]. The implication of this hypothesis is that MS patients who respond to type-1 IFNs could have a primarily Th1-mediated disease whereas patients who continue to relapse on IFN therapy may have a stronger involvement of Th17 cells in their disease pathogenesis. However, to date it has not been possible to confirm this finding in patients with MS on IFNβ treatment [133]. There are also other potential explanations for the apparent inefficacy of IFNβ in some patients with MS, including differences in genetic background and the development of neutralizing antibodies.

### 2.2. Glatiramer Acetate

Glatiramer acetate (GA) was first approved in the United States as a treatment for RRMS in 1996. GA is a polymer comprising a random sequence of the four most frequent amino acids found in MBP. GA was originally designed to induce EAE; however it was instead shown to suppress disease activity in animals [134]. Several phase III clinical trials have since demonstrated that GA administered via a daily subcutaneous injection reduces annualized relapse rate (ARR) in RRMS by approximately 30% compared to placebo [95,135,136]. GA also significantly reduced MRI-parameters of disease activity. More recent studies have demonstrated equipoise between IFNβ and GA in the treatment of RRMS [137,138]. GA has now been in routine clinical use in RRMS for over 15 years. It is generally well tolerated with no significant long-term adverse events identified.

GA is likely to exert a broad immunomodulatory effect by influencing cells of both the innate and adaptive immune system. Early studies in EAE and MS suggested that GA could influence the CD4^+^ T-cell response by switching from a pro-inflammatory Th1-type to an anti-inflammatory Th2 response [139]. This in part may be mediated via a direct effect of GA on cytokine expression profiles by dendritic cells, without affecting dendritic maturation or immunostimulatory potential [140]. A recent study has similarly shown reduced numbers of pro-inflammatory Th17 cells in the brains of GA-treated mice [141]. However, modulation of T-helper cell responses is likely to only partially explain the benefit of GA in RRMS given GA-treated mice deficient in several prominent Th2 cytokines continue to show a reduction in EAE disease activity [142]. One group has demonstrated that GA upregulates the suppressor/cytotoxic activity of GA-reactive CD8^+^ regulatory T-cells thereby potentially removing proinflammatory CD4^+^ T-cells [143,144]. It has also been suggested that GA may exert its beneficial effects in EAE and MS by restoring the function of T-reg cells [139].

In addition to effects on T-cells, GA has also been reported to influence B-cell function. Several groups have demonstrated that treatment with GA in EAE results in the stimulation of B-cell-expressed anti-inflammatory cytokines with a reciprocal reduction in the expression of pro-inflammatory cytokines [145,146]. It has also been postulated that GA may interfere with B-cell co-stimulatory signals required for T-cell activation thereby downregulating T-cell responses [139]. Support for a role for B-cells in mediating the beneficial effect of GA comes from studies demonstrating that purified B-cells transferred from GA-treated mice could abrogate EAE [145,147]. This effect was associated with decreased proliferation of auto-reactive T-cells, reduced Th1 and Th17 subsets, and an increase in IL-10 production [145].

There is growing evidence that type II (anti-inflammatory) monocytes may be the primary target of GA treatment [148]. Evidence for the importance of these regulatory antigen presenting cells comes from studies in EAE models demonstrating a correlation between clinical benefit in GA treated animals and the development of type II monocytes. These monocytes appear to have predominantly an anti-inflammatory phenotype with reduced proinflammatory TNF and IL-12 expression, and enhanced anti-inflammatory IL-10 and TGF-beta secretion [149,150]. In this *in vivo* study, GA-stimulated type II monocytes promoted the differentiation of naïve CD4^+^ T cells into Th2 cells and T-reg cells with a reciprocal reduction in Th1 and Th17 subsets, independent of antigen specificity. These results would support a primary role for type II monocytes in mediating the beneficial effects of GA via the manipulation of T-helper cell subsets.

Additional putative immunological targets for GA may include NK cell activity and MHC blockade [139,148]. A role for T-cell induced brain derived neurotrophic factor (BDNF) secretion following GA administration has also been described in MS, EAE and experimental cell lines [147,151–155]. Similarly to interferon, the effect of GA on neurotrophic factor expression may have significance in terms of providing neuroprotection in GA-treated patients, but dissection of anti-inflammatory and neuroprotective actions in the human disease is difficult.

### 2.3. Natalizumab

Natalizumab is a humanized monoclonal antibody that targets the alpha 4 subunit of the integrin alpha 4 beta 1 (or very late antigen-4–VLA-4) and alpha 4 beta 7 lymphocyte receptors. It was approved as a treatment for RRMS in 2007 following phase III studies demonstrating dramatic improvements in the rate of new MRI lesions and clinical relapses, as well as a reduction in the risk of developing disease progression [96,156]. Five years after its introduction, it remains the most efficacious of all approved treatments in RRMS. Unfortunately, natalizumab treatment is not without some risk. Over 200 cases of progressive multifocal leukoencephalopathy (PML) have been reported in patients treated with natalizumab [157]. Many cases have proven fatal (approximately 1/5) [158]. PML is a viral infection of the brain caused by the JC virus [159]. This infection is extremely rare except in the setting of systemic immunosuppression due to chemotherapy or infection with the human immunodeficiency virus [160]. The overall risk of developing PML in patients with MS treated with natalizumab is estimated at 1–2 per 1000 although the risk can be stratified by JC virus serology testing, previous immunosuppressant treatment and the duration of natalizumab treatment [157,161]. The highest risk (up to 1:100) occurs in patients with positive JC virus serology, previous immunosuppressant treatment and treatment with natalizumab for more than 2 years [157]. The risk of PML in JC-virus antibody negative individuals (around 50% of the population) may be as low as 1 in 11,625, although there is a 2% per annum rate of serocoversion necessitating annual repeat testing [157]. The decision to withdraw natalizumab treatment if a patient is found to be JC virus positive is not entirely straight forward given some studies have suggested a possible rebound phenomenon, with increased MRI disease parameters following treatment withdrawal [162] or at least a return of prior disease activity. A recent long-term study of 23 patients failed to find any evidence for a rebound phenomenon in the 14-months following withdrawal of natalizumab [163].

Natalizumab binds to the alpha 4-integrin subunit on lymphocytes, thereby blocking the interaction between the VLA-4 receptor and the VCAM-1 ligand located on cerebral endothelial cells [164]. This in turn prevents the entry of T-cells into the CNS and results in decreased CNS inflammatory activity. VLA-4-ligand binding has also been shown to result in tyrosine phosphorylation and T-cell co-stimulation *in vitro*, thereby raising the possibility of additional immunological effects of natalizumab treatment [165]. Several studies have recently investigated the effects of natalizumab on immune cell composition and cytokine expression in MS patients [166–168]. Natalizumab treatment was shown to modify the cytokine milieu depending on the length of time on treatment. Both pro-inflammatory and anti-inflammatory cytokines were influenced by natalizumab treatment [166]. It has been demonstrated that there is an increased percentage of activated leukocytes producing proinflammatory cytokines following natalizumab treatment in MS patients [168]. The authors of this work hypothesized that this effect was due to sequestration of activated cells in the peripheral circulation [168]. Other groups did not see changes in the proportions of CD4^+^ and CD8^+^ T-cells. However, there was an increased proportion of NK cells, haematopoietic stem cells and CD20^+^ B-cells after natalizumab treatment [167]. In contrast, the proportion of monocytes in peripheral blood was reduced. The percentages of T-cells, B-cells and monocytes expressing VLA-4 were all decreased. There was no effect of natalizumab on regulatory T-cell function [166–168].

Given the recent interest in NK cells as potential mediators in inhibiting CNS inflammation in MS patients treated with daclizumab, the observation that natalizumab increases the proportion of peripheral NK cells raises the possibility that this effect may play a role in the efficacy of natalizumab in RRMS. The effect on monocytes may also contribute to the therapeutic effect of natalizumab given monocytes have been implicated in both initiation and progression of lesional CNS inflammation in MS [169].

### 2.4. Fingolimod

Fingolimod is a lysophospholipid derived from an ascomycete fungal metabolite. It originally showed promise when used in models of organ transplantation. The drug progressed to phase III human trials in renal transplant patients where it failed to demonstrate superior efficacy in preventing transplant rejection when used in combination with cyclosporin A [170]. Despite a lack of immunosuppressive effects, fingolimod was noted to have effects on lymphocyte trafficking. This finding was exploited in studies of EAE models of MS where it was demonstrated that fingolimod could significantly reduce disease activity [171–173]. Results from two phase III trials (FREEDOMS and TRANSFORMS) confirmed a significant reduction in ARR, new or enhancing MRI lesions, disability progression and cerebral atrophy in RRMS patients treated with fingolimod compared to placebo or patients treated with standard dose IFNβ-1a [97,98]. These findings led to fingolimod being approved as the first oral treatment for MS in 2011. The oral route of administration for fingolimod has obvious significant advantages over the other currently available agents.

Fingolimod has unique molecular targets when compared to other MS treatments. Once ingested, the rapidly phosphorylated active form interacts with the G-protein-coupled sphingosine-1-phosphate (S-1-P) receptors 1-, 3-, 4- and 5-. Exactly how the downstream effects lead to immunomodulation in MS remains to be determined. What has been established is that binding of phosphorylated fingolimod to S-1-P1 receptors results in internalization and eventual degradation of the receptor on lymphocytes [170,174]. This in turn inhibits the egress of lymphocytes out of lymph nodes, which is likely to reduce the transmigration of T-lymphocytes into the CNS. Detailed studies have suggested that naïve T-cells, central memory T-cells and, to a lesser extent, B-cells are sequestered within secondary lymphoid tissue, whereas terminally differentiated effector memory T-cells are largely unaffected by fingolimod [175]. It has been shown that the majority of T-cells found in the CSF of MS patients are central memory T-cells with a relative depletion of effector T-cells [176]. Furthermore, Th17 cells, implicated as the primary inflammatory executors in MS, are defined by a central memory T cell phenotype and hence are sequestered in secondary lymphoid tissues following fingolimod treatment [175]. It follows then that the inability of Th17 central memory T-cells to transmigrate from the periphery to the CNS could at least partially explain the significant reduction in CNS inflammation seen following fingolimod treatment. Fingolimod can also inhibit the egress of B-cells from secondary lymphoid tissue although the effect is more modest compared with T-cells [174]. The clinical significance of B-cell sequestration in secondary lymphoid tissue remains to be determined, but it is clear that one or more of the cell populations inhibited from lymph node egress by fingolimod could be central to MS pathogenesis.

In addition to its immunomodulatory effects, fingolimod may have a direct effect on the CNS. Fingolimod can easily cross the blood-brain barrier and penetrate the CNS where it is able to bind to S-1-P receptors found on a broad range of CNS cell types [170]. In an EAE model of MS, depleting S-1-P1 from astrocytes whilst retaining peripheral receptor expression resulted in a loss of fingolimod efficacy as well as a reduction in MS-disease activity [177]. Reduced disease activity was associated with improvements in the degree of demyelination, axonal loss, and astrogliosis. Indeed, the unique mechanism of antagonizing astrocyte S-1-P1 signaling by Fingolimod may help explain the improvement in cerebral atrophy scores relative to comparator treatments observed during the FREEDOMS and TRANSFORMS trials, and rarely seen with other MS treatments.

The good short-term safety profile of fingolimod has been postulated to be due to the preservation of effector T-cell functions as well as maintaining immunosurvelliance by B-cells, CD8 T cells, NK cells and macrophages although long-term safety data is pending [170].

## 3. Emerging Disease Modifying Treatment in RRMS

### 3.1. Dimethyl Fumarate (BG-12)

In recent years there has been a shift in focus in MS treatments from parenteral treatment to oral therapy in an attempt to improve patient acceptance and compliance. In addition, there has been a growing recognition of the importance of treatments that can influence the neurodegenerative component of MS given that it is this aspect that correlates best with permanent disability. An emerging oral MS treatment is showing promise as a potential modulator of both the inflammatory and neurodegenerative components of MS; fumaric acid is an intermediate product of the citric acid cycle, a process critical for the generation of adenosine triphosphate (ATP) and the maintenance of cellular functions. Fumaric acid esters such as dimethylfumarate (DMF) and monomethyl fumarate (MMF) administered orally have been used in the treatment of psoriasis for decades in German-speaking countries [178], although the mechanism of action is not completely understood.

Studies have demonstrated a role of fumaric acid esters in reducing oxidative stress in inflammatory disease such as psoriasis [179]. This effect appears to be largely mediated via a reduction in the nuclear translocation of nuclear factor kappa-light-chain enhancer of activated B cells (NF-κB). NF-κB plays an important role as a transcription factor for a variety of pro-inflammatory mediators such as cytokines, chemokines and adhesion factors [180]. A reduction in NF-κB activity leads to the decreased expression of an array of proinflammatory molecules, which in turn can have dramatic effects on subsets of inflammatory cells.

Psoriasis has long been recognized as a Th1-mediated disease [181]. Early studies in patients with psoriasis treated with fumaric acid esters demonstrated a shift in the cytokine profile to favor the anti-inflammatory Th2 cytokines rather than the pro-inflammatory Th1 pattern [182]. Other groups have shown that DMF can decrease expression of the anti-apoptotic molecule B-cell lymphoma 2, thereby promoting apoptosis in activated T-cells and depleting T-cells [183]. Other proposed mechanisms of action for fumaric acid esters include modulation of B-cell apoptosis, reduced chemokine and adhesion molecule expression and upregulation of monocyte superoxide anion production [179].

Fumaric acid esters have been trialed in animal EAE models. DMF and MMF administered early in MOG-induced EAE resulted in reduced disease activity [184]. These findings correlated with a marked reduction in CNS macrophage infiltration. However, apart from a mild increase in IL-10 levels in treated animals, the pattern of cytokine production did not clearly favor a specific T-helper subset. One study investigated the effect of DMF in the chronic phase of MOG-induced EAE [185]. Unlike some models of EAE, the MOG-induced EAE animals develop neurodegenerative features. DMF was shown to ameliorate the disease course and protected oligodendrocytes, myelin, axons and neurons from oxidative stress. This effect was found to be dependent on the intact function of nuclear-factor (erythroid derived 2) related factor-2 (Nrf2). The Nrf2 transcriptional pathway is known to play a major role in cellular protection against oxidative stress.

The importance of Nrf2 in mediating the beneficial effect of fumaric acid esters has been confirmed in a recent study [186]. In this study, DMF or MMF treatment increased Nrf2 activity with a corresponding increase in cellular redox potential, glutathione levels, ATP levels, and mitochondrial membrane potential in a concentration-dependent manner [186]. Additional studies have also implicated increased levels of the anti-inflammatory protein haem oxygenase-2, a reduction in nitric oxide synthase 2, and suppressed nitrite production as downstream effects of fumaric acid esters [187].

The action of fumaric acid esters in providing protection from oxidative stress related death and damage to CNS cells represents a novel potential mechanism of action for MS treatment. These preliminary studies identifying both anti-inflammatory and neuroprotective effects have led to the use of BG-12, a second generation fumaric acid derivative that contains DMF, in several human clinical trials. BG-12 has now progressed to phase III trials in RRMS patients following encouraging results in phase II studies. Preliminary results from the phase III DEFINE study showed a 49% reduction in relapses in patients treated with twice daily BG-12 dosing compared with placebo over 2-year [100]. In addition, there was a 90% reduction in the number of GAD enhancing lesions in treated patients with a similar reduction in the rate of new or enlarging T2 lesions. There was a 38% reduction in 3-month confirmed disability progression. Results from the randomized CONFIRM trial containing placebo, BG-12 and GA treated groups are due to be reported shortly. Preliminary results presented in abstract form showed a relative risk reduction in ARR rate of 44% in patients treated with twice daily BG-12 and 29% in patients treated with GA [99]. Up to 12% of patients treated with BG-12 discontinued treatment mostly due to gastrointestinal upset or facial flushing [101]. A significant benefit of BG-12 is that long-term safety data is available. No obvious safety signals have been raised with long-term treatment of BG-12 or other fumarate derivatives in psoriasis patients since their first utilization in Germany in the 1950s [188] and more widespread use from 1994 [178].

### 3.2. Teriflunomide

The oral agent teriflunomide is the active metabolite of leflunomide, a treatment with recognized efficacy and relative safety in the management of rheumatoid arthritis (RA). Teriflunomide primarily acts via the inhibition of the mitochondrial enzyme dihydroorotate dehydrogenase (DHODH) [189]. DHODH is the rate-limiting enzyme in de novo pyrimidine synthesis, a process required for DNA synthesis and other metabolic functions in rapidly proliferating cells. Resting lymphocytes or homeostatically expanding lymphocytes are exempt from the effects of teriflunomide due to the presence of a DHODH-independent salvage pathway. Teriflunomide therefore is able to abrogate exaggerated immune responses such as the effector functions of activated T-cells, whilst maintaining homeostatic cellular processes [189]. *In vivo* and *in vitro* studies in mice have demonstrated that teriflunomide treatment promotes Th2 cell differentiation and enhances anti-inflammatory Th2 effector functions whilst inhibiting the proliferation and function of pro-inflammatory Th1 cells [190]. Members of the innate immune system are not excluded from the effects of teriflunomide with studies showing effects on adhesion, migration and effector functions of neutrophils and macrophages [189].

Teriflunomide may have additional effects on cellular function, independent of the inhibition of pyrimidine synthesis. *In vitro* studies using Jurkat and CTLL-4 cells have demonstrated the inhibition of tyrosine kinase pathways following teriflunomide administration [191]. The clinical significance of these effects remains to be determined. Teriflunomide has also been shown to affect multiple T-cell processes including TCR-mediated calcium mobilization, integrin avidity and intracellular adhesion molecule 1 mediated co-stimulation, and the interaction of T-cells with antigen presenting cells to form an immunologic synapse which is required for activation and further differentiation of T-cells [189,192].

Effects on T-cell migration have also been postulated [192,193]. As with other MS treatments, teriflunomide first showed promise in EAE disease models. Teriflunomide-treated animals demonstrated reduced disease severity with improvements in CNS inflammation, demyelination and axonal loss [193,194]. Results from the phase III TEMSO trial demonstrated a 31.5% reduction in ARR with high dose teriflunomide compared with placebo [102]. In this study, teriflunomide also improved MRI parameters and reduced the rate of disability progression compared to placebo. Treatment was well tolerated with the most frequently reported side effects being gastrointestinal tract upset and elevated liver enzyme levels. The results from additional phase III active comparator trials are awaited. Preliminary results from a phase II study of teriflunomide as adjunctive treatment in patients receiving IFNβ or GA have been announced [189]. The addition of teriflunomide significantly improved MRI markers of disease severity without causing an increase in adverse events. Teriflunomide may prove to be a useful add-on treatment in those patients in whom monotherapy with more conventional MS therapies provides inadequate disease control.

### 3.3. Laquinimod

Laquinimod, or quinolone-3-carboxamide, is a novel orally active drug that is showing promise in the treatment of RRMS. Laquinimod represents a chemically modified derivative of roquinimex, an immunoregulatory molecule that was investigated in the mid-1990’s as a treatment for RRMS but was subsequently withdrawn from further development due to toxicity concerns (pericarditis, pleuritis, chest pain and myocardial infarction) and 2 deaths [195–197]. In EAE models of MS, laquinimod was shown to attenuate disease with improvements in both CNS demyelination and chronic axonal loss [198–200]. These findings raised the possibility that laquinimod may be beneficial in reducing both the acute inflammatory phase and the chronic neurodegenerative component of MS. A phase III study comparing laquinimod to placebo in RRMS has recently been published [103]. This study showed a non-significant 23% reduction in ARR together with a small, but statistically significant improvement in disease progression in patients treated with laquinimod compared with placebo. MRI lesions were similarly significantly reduced in laquinimod-treated patients. Treatment was well tolerated with transiently elevated liver enzyme levels being the most commonly reported adverse event. In a prespecified exploratory analysis, laquinimod-treated patients had less brain-volume loss compared to patients receiving placebo. Together with the improvement seen in disability progression, these results support the findings from EAE studies, and raise the possibility that laquinimod may be beneficial in reducing the neurodegenerative component of MS.

Laquinimod is rapidly absorbed following oral administration with low levels able to be detected within the CNS within hours [201]. Several studies involving EAE models have shown that laquinimod decreases pro-inflammatory cytokines [198] and promotes a deviation from the pro-inflammatory Th1 pattern to the anti-inflammatory Th2/Th3 cytokine milieu [200,202]. In particular, levels of IL-17, previously implicated as a key component in promoting CNS inflammation in MS, are significantly reduced following laquinimod treatment [198]. These effects on inflammatory cells do not interfere with antigen-induced T-cell proliferation [200] nor do they affect the ability of animals to mount a cellular or humoral immune response [201] This is despite a recent publication suggesting laquinimod results in the suppression of genes related to antigen presentation and corresponding inflammatory pathways in peripheral blood mononuclear cells from MS patients [203]. Several groups have also demonstrated that laquinimod reduces leukocyte migration into the CNS in EAE [198,199,201]. One study has suggested that laquinimod reduces the ability of VLA-4 to integrate chemokine signals and to generate high binding affinity for VCAM-1, a process necessary for effector T-cells to traverse the blood brain barrier and enter the CNS [198].

In addition to an immunomodulatory mechanism, laquinimod may act directly in the CNS to reduce demyelination and axonal damage. In both animal and human MS subjects, treatment with laquinimod resulted in significantly higher levels of serum BDNF compared with placebo-treated subjects [204]. BDNF is one of a family of neurotrophins that function as important regulators of neuronal and axonal survival. Mice deficient in CNS-derived BDNF have a more severe course of EAE and increased axonal loss [205]. A conditional BDNF knockout model showed that only loss of BDNF expression during disease initiation results in a more severe disease phenotype with loss of axonal integrity [206]. It is appealing to consider that early treatment with laquinimod may confer a degree of neuroprotection in MS patients via an elevation in neurotrophic factor levels.

### 3.4. Alemtuzumab

Alemtuzumab is a humanized monoclonal antibody that targets CD52, a glycoprotein cell surface marker of unknown function found on all differentiated lymphocytes and monocytes but not on hematopoietic precursors [207]. Alemtuzumab is an approved treatment for chronic lymphocytic leukaemia and T-cell lymphoma, and is a conditioning treatment prior to renal or bone marrow transplantation. For the treatment of MS alemtuzumab is administered as a course of infusions over 5 consecutive days followed by a second course over 3 days 12 months later [208]. The need for any further courses of treatment remains to be seen but appears to be low [208,209]. Treatment with alemtuzumab results in the rapid depletion of CD52-expressing cells via antibody-dependent cellular cytotoxicity. Restoration of the immune cell population occurs over a period of months to years. Overall B-cell and monocyte counts recover within 3-months, with an overcorrection of B-cells to 165% of baseline up to 12-months post-treatment likely mediated by a surge in B cell activating factor belonging to the TNF family (BAFF) [210]. This supra-normal B-cell response may be responsible for the increased rates of autoimmune disease seen as a complication of alemtuzumab-treatment in MS clinical trials (see below). In contrast, memory B-cells remain suppressed, reaching only 25% of baseline at 12-months. This in part may be explained by the lack of T-cell help required for B-cell development. CD8^+^ T-cells require over 2-years and CD4^+^ T-cells over 5-years to return to baseline following treatment [211]. An exception to this is the enrichment of the T-cell pool with memory T-cells that have a regulatory phenotype noted within the first 6-months post-treatment [211].

Alemtuzumab was first trialed in a small group of MS patients in the early 1990s. The treatment dramatically abolished acute relapses and reduced accumulated disability in RRMS patients, but failed to slow disability in the one third of patients with progressive disease [209]. The authors concluded that alemtuzumab was effective against acute inflammatory disease. However, any effect on disability progression required treatment administered prior to the onset of the progressive phase of MS. Since this time, results from a phase II trial comparing alemtuzumab with IFNβ-1a have been published [212]. The CAMMS-223 trial enrolled RRMS patients with a disease duration of less than 3 years and an expanded disability status scale (EDSS) less than 3. Alemtuzumab treatment reduced MS relapses by 74% compared with IFNβ-1a treatment. Furthermore, alemtuzumab reduced the risk of disability progression by 71%, with patients’ EDSS improving by 0.39 points at 3 years. This is compared to a worsening of EDSS by 0.38 points in patients treated with IFNβ-1a. The enthusiasm for alemtuzumab treatment was somewhat tempered by the observation that up to 30% of alemtuzumab-treated patients developed immune-mediated diseases such as autoimmune thyroid disease and idiopathic thrombocytopaenic purpura (ITP). Indeed, one patient in the CAMMS-223 trial died of a brain haemorrhage after developing alemtuzumab-induced ITP. A study has demonstrated that patients who developed an autoimmune complication with alemtuzumab treatment had significantly higher levels of IL-21 and lower levels of IL-7 [213]. These biomarkers may prove useful in predicting the patients who are most likely to develop these autoimmune complications.

Results from two phase III trials involving alemtuzumab have recently been presented [104,105]. Both trials compared the efficacy of alemtuzumab against IFNβ-1a in the treatment of RRMS. CARE-MS 1 patients were treatment naïve whereas CARE-MS II patients were required to have relapsed on previous treatment to be eligible. Both studies showed a superiority of alemtuzumab over active comparator in terms of relapse reduction and MRI criteria. Only CARE-MS II reproduced the improvement in disability progression seen in the phase II study of alemtuzumab. Despite a lack of benefit seen in EDSS-based accumulated disability measures for the CARE-MS I trial, the study did show improvement in cerebral atrophy, an MRI parameter known to correlate with long-term disability.

The observation that a highly effective MS treatment can induce other autoimmune diseases is of some interest for hypotheses of MS pathogenesis. Most of the alemtuzumab-induced autoimmune diseases are antibody-mediated, suggesting that MS is unlikely to be an autoantibody-mediated disease.

### 3.5. Daclizumab

Daclizumab is a humanized monoclonal antibody directed against the IL-2 receptor (CD25) alpha chain. Daclizumab binds to the IL-2 binding site of CD25 thereby masking the binding site without influencing signaling activities and without instigating cytotoxicity [214]. The interaction of IL-2 with its receptor is the major autocrine growth factor pathway important for expanding effector and regulatory T-cells [215]. CD25 forms part of the high affinity IL-2 receptor which is expressed at low levels on resting T-cells but is rapidly upregulated following T-cell activation. Daclizumab was thus originally designed to treat diseases mediated by activated T-cells, such as HTLV-I-induced adult T cell leukaemia. It was subsequently found to be beneficial in preventing rejection of solid organ transplants and in the treatment of inflammatory uveitis [216–220]. Daclizumab has recently been shown to be efficacious in RRMS. A phase IIb trial (CHOICE study) randomized patients with RRMS to IFNβ-1a plus low-dose or high-dose daclizumab or placebo. Patients receiving the high dose daclizumab had a 72% reduction in new GAD lesions compared to patients receiving placebo [221]. Importantly, daclizumab treatment was not associated with an increased risk of serious adverse events, although there was an infection related death in the treatment arm of the SELECT phase III trial [222].

Treatment with daclizumab increases the frequency of NK cells [221,223–225]. This expansion of NK cells is thought to be due to the increased bioavailability of IL-2 to NK cells which in turn stimulates NK cell proliferation through binding to the IL-2 intermediate affinity receptor [223]. Additional mechanisms may include IL-2-induced differentiation of NK cell pre-cursors and/or reduced apoptosis of NK cells [226]. In most studies, daclizumab treatment appears to selectively target the expansion of NK CD56^+^ bright cells. Several groups have demonstrated that MS patients who only partially responded to daclizumab did not show expansion of this cell population raising the possibility that CD56^+^ bright cells may be a useful biomarker for response to treatment [221,225]. In contrast, others have shown that both NK CD56^+^ bright and NK CD56 dim cells are required for disease protection in a human-mouse chimera model [224]. In this model, disease protection was conferred by CNS-resident NK cells. The group proposed that the mechanism of action of NK cells in reducing disease activity centered on reducing the capacity of microglia to stimulate Th17 responses [227]. Many other putative mechanisms have been suggested to explain the efficacy of daclizumab in MS. These include NK cell-mediated cytotoxicity of CD4^+^ T-cells through a perforin/granzyme-independent mechanism [228,229], interference with early myeloid dendritic cell-T-cell interaction [229], and impairment of effector T-cell function via blockade of CD40L expression [230], to name a few.

### 3.6. B-Cell Therapies

#### 3.6.1. Rituximab

Rituximab is the first genetically engineered chimeric mouse-human monoclonal antibody designed to target B-cells expressing the CD20 trans-membrane protein. Stem cells, pro-B cells and differentiated plasma cells are exempt from the effects of rituximab due to the fact that they do not express CD20. Rituximab efficiently removes CD20^+^ B-cells from the circulation and as such it has proven beneficial in the treatment of B-cell lymphoma. Rituximab lyses B-cells via complement-dependent cytotoxicity, antibody-dependent cytotoxicity and B-cell apoptosis [231]. From its inception as an approved treatment for non-Hodgkin B-cell lymphoma in 1997, rituximab has also been trialed in a number of autoimmune and inflammatory conditions including RA, chronic lymphocytic leukaemia, ITP, NMO, myasthenia gravis and systemic vasculitides, to name a few [232]. In general rituximab is well tolerated with the most common reported adverse effect being infusion reactions. Unfortunately a number of cases of PML have been identified in patients receiving rituximab [233–235], reminding us that treatments with improved efficacy can carry significant clinical risk.

Rituximab has now been investigated in a number of clinical trials in MS. A phase II trial involving 104 patients with RRMS compared a single course of rituximab to placebo over 48 weeks. The study demonstrated that rituximab treatment significantly reduced MRI parameters of disease activity and relapse rates compared to placebo [74]. A phase I study looking at the effect of two courses of rituximab 6 months apart and followed for 72-weeks was also published in 2008. This study found a similar reduction in new GAD-enhancing lesions as well as an apparent reduction in relapses compared to the year before therapy [75]. The efficacy of rituximab as an add-on therapy was assessed in a phase II study involving 30 patients with RRMS who continued to have relapses despite treatment with injectable disease modifying therapy [76]. Rituximab add-on therapy resulted in a significant improvement in radiological endpoints as well as an improvement in the MS functional composite. Disappointingly, a phase II/III placebo-controlled trial of rituximab in PPMS failed to show a significant increase in the time to confirmed disability progression in rituximab-treated patients [236]. However, subgroup analysis showed younger patients and those with GAD-enhancing lesions had significantly longer time to confirmed disability progression [236]. Overall rituximab was well tolerated with no serious adverse events reported. A recently published Cochrane review assessing the evidence for rituximab in RRMS concluded that any potential benefits remain inconclusive due to high attrition bias, low patient numbers and short-term follow-up of trial participants [237].

Rituximab rapidly depletes circulating B-cells within 2-weeks of treatment [75]. Total circulating antibody levels in serum and CSF are unaffected by rituximab [75,238]. This observation, together with the rapidity of action of rituximab, would argue against a reduction in pathogenic autoantibodies as the mechanism of action. Alternative mechanisms have been hypothesized including a reduction in antigen presenting B-cell activity, decreased B-cell co-stimulatory signaling and the modulation of B-cell mediated pro-inflammatory cytokine production responsible for “bystander activation” of T-cells [78,231]. A reduction of IL-6 secretion from B-cells has been suggested as a major mechanism of action for CD20-depleting treatment [239]. In both murine EAE and RRMS patients treated with CD20^+^ B-cell depleting therapy, decreased IL-6 levels were associated with a reduced Th17 response [239]. An unexpected finding arising from analysis of patients enrolled in the Phase II rituximab add-on study was that treated patients showed a 50% reduction in CSF T-cells in addition to the depletion of B-cells [238]. The authors attribute at least some of this effect to a reduction in the CSF levels of two chemokines, C-X-C motif chemokine receptor ligand 13 and C-C motif chemokine ligand 19, involved in the CNS trafficking of lymphocytes [238].

#### 3.6.2. Ocrelizumab

Following the publication of positive results from early trials of rituximab in the treatment of MS, several other B-cell depleting therapies have entered clinical trials. Ocrelizumab is a recombinant humanized monoclonal antibody that also selectively targets CD20^+^ B-cells. It shares a significant degree of sequence homology with rituximab, however it binds to a distinct but overlapping region of CD20 [240]. It is reportedly associated with greater antibody-dependent cytotoxicity and less complement-mediated cytotoxicity compared with rituximab [241]. Importantly, ocrelizumab is likely to be less immunogenic and cause less infusion reactions than rituximab given it is a fully humanized antibody.

A phase II placebo-controlled study of ocrelizumab in RRMS showed a 96% reduction in GAD-enhancing lesions in the highest dose group compared to placebo [241]. All patients receiving ocrelizumab had improved MRI parameters of disease activity compared to patients treated with interferon beta-1a. Reduced ARR was also seen in ocrelizumab treated patients. Six patients withdrew in the first 24-week of the study due to safety concerns. One patient receiving high-dose ocrelizumab died due to a systemic inflammatory response syndrome. Concerning safety signals have arisen from phase III trials of ocrelizumab in the treatment of RA. A significant number of ocrelizumab-treated patients experienced serious and opportunistic infections [242,243]. Most of these adverse events were in patients recruited from Asia and in patients receiving concomitant immunotherapy. As a result of safety concerns, all trials of ocrelizumab in the treatment of RA have been discontinued.

#### 3.6.3. Atacicept

Atacicept is a novel therapy also designed to target B-cells in autoimmune disease. Atacicept is a human recombinant fusion protein comprising the binding region of a receptor that binds the cytokines A proliferation-inducing ligand (APRIL) and B lymphocyte stimulator (BLyS). These cytokines are members of the TNF superfamily and are expressed by cells of both the innate and adaptive immune systems. BLyS and APRIL are involved in the regulation of B-cell development, homeostasis and survival [244]. They can form homo- and hetero-trimers that have unique binding affinities to three receptors; TACI, BCMA and BAFF-R [244]. Increased BLyS and APRIL expression have been associated with a number of autoimmune-mediated diseases including MS [245–248]. Overexpressing BLyS in transgenic mice results in the expansion of B-cells and effector T-cells. These mice not surprisingly develop autoimmune-like manifestations characterized by the production of autoantibodies and immune complex deposition [249]. Inhibiting BLyS and APRIL signaling therefore offers a potentially novel therapeutic target in the treatment of autoimmune disease.

Atacicept acts as an antagonist, blocking the binding of both APRIL and BLyS to their receptors and inhibiting the downstream effects on B-cells. Due to the variable expression of the receptors in B-cell subsets, atacicept affects mature B cells and antibody producing plasma cells but spares B-cell progenitors and memory B-cells [245,250]. Pre-clinical studies confirmed the expected reduction in circulating B-cells and immunoglobulin levels in atacicept-treated animals [251]. No significant safety signals emerged from phase I studies of atacicept in systemic lupus erythematosus and RA [244]. Despite encouraging findings in animal models of EAE, a phase II study of atacicept in patients with RRMS was suspended prematurely after researchers found an early increase in CNS inflammatory activity in atacicept-treated patients.

This unexpected outcome is disappointing in light of the previously reported beneficial effects of B-cell targeted therapy in MS. Unfortunately, data from the phase II atacicept trial has not been published therefore further information is currently unavailable. Interestingly, a phase II/III trial of atacicept in combination with mycophenolate mofetil and corticosteroids in the treatment of lupus nephritis was also terminated prematurely after the enrolment of just six patients due to unexpectedly low immunoglobulin levels associated with serious infections [252]. Furthermore, a recently published study demonstrated that atacicept did not meet the primary end-point in phase II trials in RA despite expected biological effects [253].

The increased CNS inflammatory activity seen in atacicept-treated RRMS patients is intriguing and emphasizes the complex role of B-cells in MS. Unlike other B-cell treatments, atacicept only affects mature B-cells and antibody-secreting plasma cells, sparing progenitor cells and memory B-cells. Phase I studies of atacicept in RA demonstrated a biphasic effect on B-cells, with an initial dose-related increase in B-cell numbers within 2-weeks of the first dose of atacicept, largely attributed to a transient increase in memory B-cells [254]. Could this surge in memory B-cells be somehow responsible for mediating early CNS demyelination? A recent study demonstrated that the memory B-cell pool in some RRMS patients can harbor MBP and MOG specific B-cells that can activate T-cells and cytokine secretion [255]. Increased memory B-cells following atacicept treatment could theoretically result in enhanced neuro-antigen reactive T-cell proliferation and inflammatory cytokine secretion leading to increased CNS demyelination.

It is increasingly recognized that B-cells can have divergent immunomodulatory roles with both pro-inflammatory and anti-inflammatory functions. B-cell depleting therapies therefore could have unexpected results, including pro-inflammatory responses, if anti-inflammatory (B-reg) functions are affected. An alternative explanation for the increased CNS demyelination seen in atacicept-treated RRMS patients could involve the rapid reduction of immunoglobulin levels seen after atacicept treatment. There is some evidence from animal models that CNS autoantibodies can promote remyelination and support neurite extension [59]. Atacicept therefore may reduce protective CNS autoantibodies involved in myelin repair resulting in increased CNS demyelination.

The failure of atacicept in the treatment of RRMS has the potential to provide us with further insights into the pathophysiology of MS. At the very least, it has reinforced the complex nature of the immune system in the causation of MS.

## 4. Conclusions

Despite decades of research, current treatments for RRMS are unable to induce complete remission in all patients, and have less than optimal impact on disease progression. An individual’s response to a specific treatment is highly variable and is likely to reflect heterogeneity in terms of the underlying immunopathogenesis. What has been established is that all current or emerging treatments in RRMS have widespread, and often unexpected, effects on the immune system. Targeting CD4^+^ cells, either via the manipulation of T-helper subsets or through T-cell depletion, appears to be a common theme amongst most MS treatments. What is perhaps more surprising is the potential effect of some treatments on CD4^+^ T-reg cells and NK cells. The finding that daclizumab results in the expansion of NK cell populations, and that this effect correlates with a clinical response, was unexpected given that this monoclonal antibody was designed to inhibit the activity of effector T-cells [221,225]. Several studies have subsequently shown that other RRMS treatments, such as natalizumab, IFNβ and GA, may all exert beneficial effects through the upregulation of NK cells [119–122,139,148,167]. Similarly, increased T-reg activity has been described in response to a number of MS treatments. These findings should encourage researchers to revisit the possibility that NK cell and T-reg dysfunction may make an important contribution to the immunopathogenesis of MS.

Perhaps most surprising is the variable effect of treatments designed to inhibit B-cell activity. The concept that B-cells are integral to the CNS inflammation in MS has been fuelled by recent studies showing clonally expanded B-cell aggregates populating the meninges and parenchyma in patients with MS. Positive results from trials with rituximab in RRMS support a key role for B-cells in promoting disease activity in MS. It remains to be determined whether or not the action of Rituximab is mediated through a reduction in the production of autoantibodies or the withdrawal of the antigen presentation and cytokine release functions of B-cells. IFNβ, GA, fingolimod and alemtuzumab have all been shown to affect B-cell function, with evidence that IFNβ and GA can modulate cytokine secretion from B-cells with downstream anti-inflammatory effects on T-cell subsets [129,145]. In contrast, the observation that atacicept appeared to promote CNS demyelination forced researchers to re-evaluate their original hypothesis regarding B-cells. Whilst the exact reasons for the lack of efficacy of atacicept in MS remain unclear, it is likely that B-cells have divergent roles in MS. So-called “pathogenic” B-cells may drive CNS inflammation through both antibody-dependent and independent mechanisms, whilst “regulatory” B-cells may help downregulate inflammatory pathways and promote remyelination and repair.

The relative success of MS treatments targeting leukocyte migration has proven to be a highlight in MS clinical research over the last decade. Whilst both natalizumab and fingolimod were designed to impede the trafficking of lymphocytes into the CNS, a number of other treatments have been shown to indirectly impair lymphocyte migration. Treatments such as IFNβ, rituximab, teriflunomide and laquinimod have all been shown to exert effects on lymphocyte migration, usually by the modulation of chemokine and adhesion molecule expression [118,192,193,198,238]. Clearly the migration of peripheral lymphocytes and monocytes into CNS compartments remains a critical step in the instigation and propagation of demyelination and tissue damage in MS.

A review of the literature surrounding MS treatments has revealed a number of additional effects which may prove to be useful targets in the development of future therapies. Several MS treatments, including laquinimod, IFNβ and GA, have been shown to increase levels of neurotrophic factors such as BDNF [126,128,151–154,204]. Neurotrophic factors are important in promoting repair and survival of neurons. It remains to be determined whether increases in neurotrophic levels translate into long-term clinical benefit. Whilst manipulating peripheral immune cells appears to be the common denominator in MS treatments, fingolimod has unique direct effects on astrocytes [177]. This somewhat unexpected effect may be responsible for improvements in cerebral atrophy scores seen in patients participating in phase III clinical trials of fingolimod. The notion of a reduction in oxidative stress through Nrf2 in the case of BG-12 is a tantalizing prospect in terms of neuroprotective mechanisms of action for MS therapy. These multimodal mechanisms also raise the possibility of combination therapies which might aim to both reduce levels of inflammation and promote the viability of CNS cells.

The pathogenesis of MS is proving to be complex, with the exact mechanisms underlying the disease process remaining elusive. Delving further into the mechanisms of action for established and emerging MS therapies has similarly highlighted a level of complexity that in all likelihood is only partially understood. Further questions arise when studies reveal disparities between *in vitro* studies, animal models of disease and clinical trials in people with MS. Despite a number of interesting aspects to the immunopathogenesis being identified as important in MS, with the exception of neutralizing antibodies which can explain breakthrough disease in some patients (IFNβ and natalizumab) and the JC virus antibody test which can stratify risk of PML in the use of natalizumab, there are currently no clinically useful predictive biomarkers to guide individualized therapy in MS.

With greater understanding of the pathogenesis of MS comes the opportunity for more targeted treatments and improved disease control. Whilst EAE models have already demonstrated their usefulness in the development of new MS treatments, they undoubtedly provide an overly simplistic view. MS is a heterogeneous disease which is likely to undergo a degree of flux in terms of which immune pathways are relevant at any particular stage of an individual’s disease. Immuno-profiling and biomarkers of disease activity may be useful in the future to help direct treatment decisions for individuals, with the ultimate aim of improving overall disease control.

## Figures and Tables

**Table 1:** Summary of MS treatments with phase III data.

Agent [ref]	Mechanism of action	Route of admin	Efficacy			Adverse effects		
			ARR	Prog	MRI	Nature	Level	Freq
**Currently approved therapies**								
β-interferon † [14,91–94]	Promotes Th2 environment Anti-inflammatory (reduced IL-17; IL-23) NK; T-reg expansion	SC/IM	+	(+)	++	Injection site reactions	+	++
						Flu-like symptoms	+	++
						Depression	+	++
						Lymphopaenia	+	+++
						LFT abnormality	+	++
						Neutralising antibodies	(+)	++
Glatiramer acetate [95]	Promotes Th2 environment Modulation of APCs Reduced TNF; IL-12	SC	+	−	+	Injection site reaction	+	+++#
						Post-injection reaction	+	+++
Natalizumab [96]	Antibody blockade of VLA-4	IV	+++	+	+++	Infusion reaction	++	++
						PML	+++	+
						Neutralising antibodies	+	+
**Fingolimod** [97,98]	Blockade of S-1-P1 receptors Trapping of lymphocytes in lymph node (Neuroprotection)	Oral	++	+	+++	Bradycardia	++	++
						Macular oedema	++	+
						LFT abnormality	+	+++
						Lymphopaenia	++	++
**Emerging therapies**								
**Dimethyl fumarate** [99–101]	Reduces oxidative stress Increased Nrf2	Oral	++	+	+++	Gastrointestinal symptoms	++	+++
						Flushing	+	+++
**Teriflunomide** [102]	Dihydroorotate dehydrogenase inhibitor Prevents pyrimidine synthesis Restricts lymphocyte proliferation	Oral	+	+	++	Paraesthesia	+	+++
						LFT abnormality	+	+++
						Gastrointestinal symptoms	+	+++
						Arthralgia	+	+++
						Alopecia	+	+++
**Laquinimod** [103]	Quinolone-3-carboxamide Promotes Th2 shift (Neuroprotection)	Oral	+	++	+	LFT abnormality	+	+++
						Arthralgia	+	++
						Back pain	+	+++
**Alemtuzumab** [104,105]	CD52 monoclonal antibody Lysis of lymphocytes	IV	+++	+	+++	Thyroid autoimmunity	++	++
						ITP	++	+
						Goodpasture’s syndrome	+++	+
						Oral/genital herpes *	+	++

APCs antigen presenting cells; ARR annualized relapse rate; IM intramuscular injection; ITP idiopathic thrombocytopaenia; IV intravenous infusion; LFT liver function tests; PML progressive multifocal leukoencephalopathy; SC subcutaneous injection; Prog- disease progression; Level severity of adverse event; Freq frequency of adverse event (see text for cytokine/receptor nomenclature); Efficacy measure: − no evidence; (+) equivocal evidence; + some benefit; ++ reasonable benefit; +++ considerable benefit; Level of adverse events: + trivial or mild (resolves with no or simple treatment); ++ significant problem (requires withdrawal of therapy or specific treatment; potentially harmful); +++ likely to be harmful/fatal; Frequency: +: <1%; ++: 1–10%; +++: > 10%;

† frequencies of β-interferon adverse effects vary according to dose; frequency and route of administration;

# although seen in 15% of patients; generally an infrequent event;

* can be abrogated by pre-treatment with acyclovir.
